# A cassava common mosaic virus vector for virus-induced gene silencing in cassava

**DOI:** 10.1186/s13007-021-00775-w

**Published:** 2021-07-12

**Authors:** Decai Tuo, Peng Zhou, Pu Yan, Hongguang Cui, Yang Liu, He Wang, Xiukun Yang, Wenbin Liao, Di Sun, Xiaoying Li, Wentao Shen

**Affiliations:** 1 Key Laboratory of Biology and Genetic Resources of Tropical Crops, Ministry of Agriculture and Rural Affairs & Institute of Tropical Bioscience and Biotechnology, Chinese Academy of Tropical Agricultural Sciences, Haikou, 571101 China; 2 Hainan Key Laboratory for Protection and Utilization of Tropical Bioresources &, Institute for Tropical Agricultural Resources, Chinese Academy of Tropical Agricultural Sciences, Haikou, 571101 China; 3 College of Plant Protection, Hainan University, Haikou, 570228 China; 4 College of Horticulture, Hainan University, Haikou, 570228 China

**Keywords:** Cassava, Cassava common mosaic virus, Virus vector, Virus-induced gene silencing

## Abstract

**Background:**

Cassava is an important crop for food security and industry in the least-developed and developing countries. The completion of the cassava genome sequence and identification of large numbers of candidate genes by next-generation sequencing provide extensive resources for cassava molecular breeding and increase the need for rapid and efficient gene function analysis systems in cassava. Several plant virus-induced gene silencing (VIGS) systems have been developed as reverse genetic tools for rapid gene function analysis in cassava. However, these VIGS vectors could cause severe viral symptoms or inefficient gene silencing.

**Results:**

In this study, we constructed agroinfection-compatible infectious cDNA clones of cassava common mosaic virus isolate CM (CsCMV-CM, genus *Potexvirus*, family Alphaflexiviridae) that causes systemic infection with mild symptoms in cassava. CsCMV-CM was then modified to a viral vector carrying the Nimble cloning frame, which facilitates the rapid and high-throughput cloning of silencing fragments into the viral genome. The CsCMV-based vector successfully silenced *phytoene desaturase* (*PDS*) and *magnesium chelatase subunit I* (*ChlI*) in different cassava varieties and *Nicotiana benthamiana*. The silencing of the *ChlI* gene could persist for more than two months.

**Conclusions:**

This CsCMV-based VIGS system provides a new tool for rapid and efficient gene function studies in cassava.

**Supplementary Information:**

The online version contains supplementary material available at 10.1186/s13007-021-00775-w.

## Background

Cassava (*Manihot esculenta* Crantz, Euphorbiaceae) is native to the Amazon basin in South America, and its edible starchy storage root provides a major food source for nearly a billion people in tropical and subtropical regions [1, 2]. Recent data from the Consultative Group for International Agricultural Research (CGIAR) showed that cassava is becoming the second most important food crop in the least-developed countries and the fourth most important in developing countries, with a total production of 218 MT. Over half of the total production is in Africa and another third in Asia (https://www.rtb.cgiar.org/crops/cassava/). With increasing yields and technological innovations, cassava is not only an important food security crop but also an industrial and biofuel crop for production of industrial starch and ethanol in some countries [2].

Recent advances in next-generation sequencing technology have contributed to the completion of whole-genome sequences for wild and cultivated cassava lines [3, 4]. Furthermore, large numbers of transcriptomic profiles have resulted in the identification of many candidate genes associated with cassava tissue development, metabolism, and responses to biotic and abiotic stress [5–8]. The accumulation of these genomic resources has increased the need for the development of reverse genetic technologies to identify functional genes that control desirable critical traits. The cassava genetic transformation technology is mature [9, 10], and available reverse genetic tools, such as RNA interference and gene-editing, have been used to validate gene function by the stable genetic transformation [11–14]. However, cassava transformation is a laborious and lengthy process, and the protocols are not applicable to all cassava genotypes [10]. Virus-induced gene silencing (VIGS) as a powerful reverse genetic approach is a convenient and efficient alternative to genetic transformation [15]. In recent decades, more than 50 different plant DNA and RNA viruses and their viral satellites have been developed into VIGS vectors [16]. Through VIGS, many gene functions have been elucidated, including those involved in organ development, secondary metabolism, and responses to plant biotic and abiotic stresses [16]. In cassava, two mosaic geminiviruses, African cassava mosaic virus (ACMV) and East African cassava mosaic virus (EACMV), were developed into VIGS systems that have been reported to work efficiently in cassava [17–20]. However, ACMV and EACMV can cause characteristic chlorosis and distortion in cassava leaves [15, 19]. These symptoms could interfere with the evaluation of VIGS effects and phenotypes in plants. Furthermore, viral vectors should observe strict importation and biosafety regulations [21]. The use of ACMV- and EACMV-based VIGS vectors is not permitted in certain countries, like China, because these viruses are not native to these areas and could easily cause pandemics. More recently, tobacco rattle virus (TRV, genus *Tobravirus*, family Virgaviridae)-based VIGS vector, which is widely used in Solanaceae, was used to silence the visible marker gene *phytoene desaturase* (*PDS*) in cassava, but the resulting photobleached phenotype was very weak [22, 23]. Therefore, it is necessary to exploit new and more appropriate VIGS vectors for gene function analysis in cassava.

Cassava common mosaic virus (CsCMV) belongs to the genus *Potexvirus* (family Alphaflexiviridae). CsCMV was first reported in southern Brazil and is widespread in Latin America; it has also recently been found in China [24–26]. Compared with severe chlorosis and leaf distortion caused by cassava mosaic geminiviruses, the typical symptoms of CsCMV infections are generally milder mosaics with dark and light patches in cassava leaves [24, 27]. Thus, CsCMV is a candidate for use as a VIGS vector in cassava. Like other potexviruses, CsCMV contains a monopartite positive sense, single-stranded RNA genome 6,395 nucleotides (nt) in length excluding the 3′-poly-A tail [25]. Its genome comprises five open reading frames (ORFs) encoding an RNA-dependent RNA polymerase (RdRp), three triple gene block (TGB) proteins, and a coat protein (CP). Several potexviruses, such as potato virus X (PVX), foxtail mosaic virus (FoMV), and pepino mosaic virus (PepMV), have been developed as vectors for expression of heterologous proteins [28–31] and transient loss-of-function studies based on VIGS in diverse dicot and monocot plant species [32–34].

We developed agroinfection-compatible infectious cDNA clones of CsCMV isolate CM (CsCMV-CM), which causes mild systemic symptoms in cassava. CsCMV-CM was modified to a viral vector carrying the Nimble cloning (NC) frame [35] that facilitates rapid and high-throughput insertion of gene-silencing fragments into the viral genome. We successfully silenced *PDS* and *magnesium chelatase subunit I* (*ChlI*) genes in six different cassava varieties and *Nicotiana benthamiana* using the CsCMV-based vector. This vector will provide a new tool for rapid and efficient gene function studies in cassava.

## Results

### Construction of the CsCMV-based agroinfectious clone and vector

**Fig. 1:**
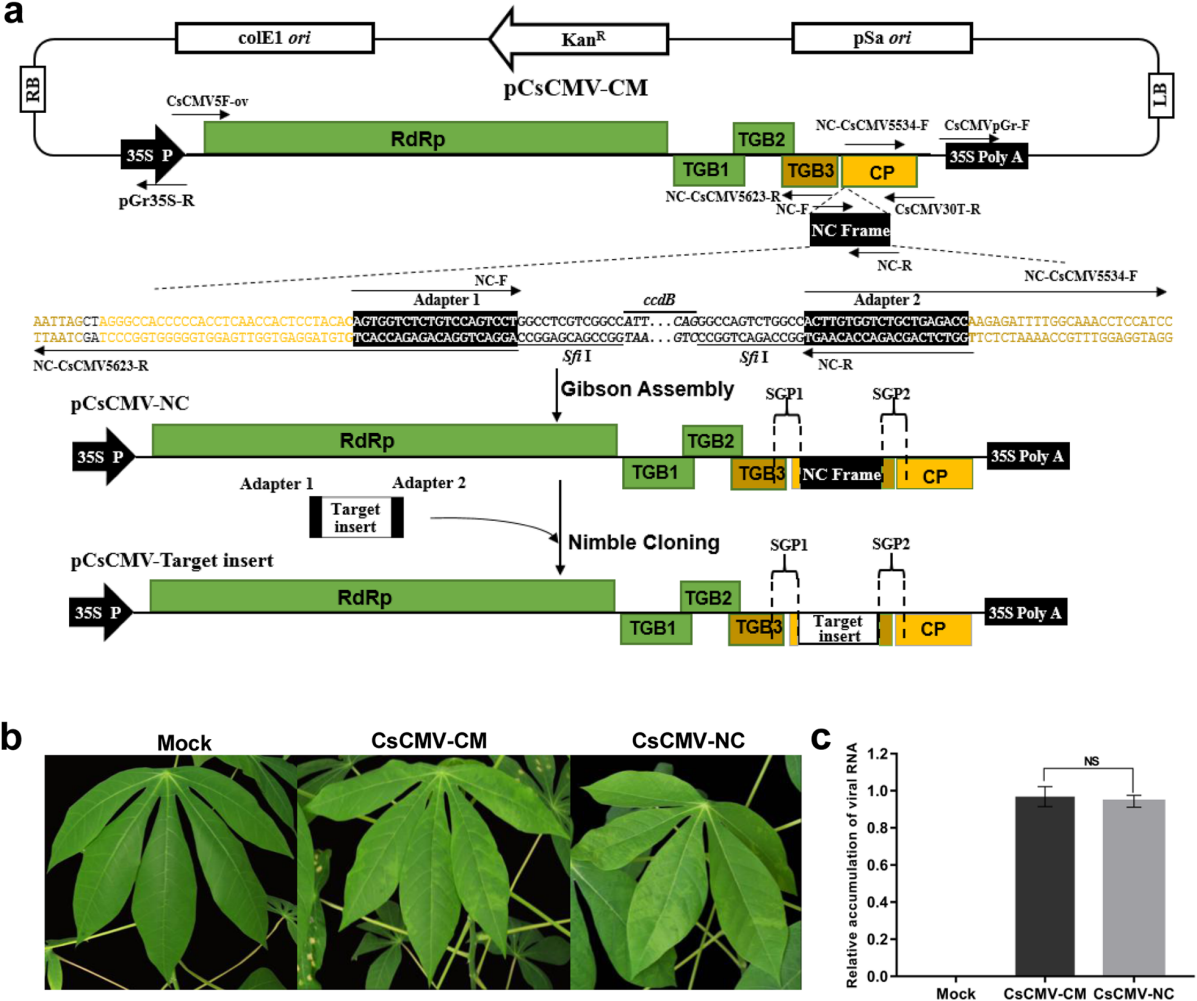
Construction and infectivity of a CsCMV-CM-based vector (pCsCMV-NC).** a** Schematic of infectious clone pCsCMV-CM and pCsCMV-NC vector. The full-length genomic cDNA of CsCMV-CM was cloned into between CaMV 35S promoter (35S P) and poly(A) signal of a T-DNA binary vector pGreenII-35S to generate pCsCMV-CM. A duplicated 90-bp putative CsCMV-CM CP subgenomic promote (SGP1) and a Nimble Cloning (NC) frame sequence (adapter 1–*Sfi* I–*ccdB* gene–*Sfi* I–adapter 2) were engineered into viral genome at upstream of the authentic CP promoter (SGP2), and the resultant vector was designated as pCsCMV-NC. The duplicated SGP includes 60 bp upstream of the CP start codon and ended 30 bp downstream (GenBank accession numbers MW175326, nt 5534–5623). The target gene fragment was flanked by adapter 1 and 2 of the NC frame and can be cloned into the pCsCMV-NC vector using Nimble Cloning. A total of 5 major open reading frames (ORFs) of the CsCMV genome are indicated by colored boxes: an RNA dependent RNA polymerase (RdRp), three triple gene block (TGB) proteins and a coat protein (CP). White rectangles and arrows indicate elements comprising the backbone of the pGreenII-35S vector. The nucleotide sequences of adapter 1 and 2 in the NC frame sequence are shaded in black. The *Sfi* I sites are underlined and the *ccdB* gene is marked in italics. Black arrows indicate primers used to construct agroinfectious clone pCsCMV-CM and pCsCMV-NC vector (Additional file 2: Table S2). **b** Systemic symptoms induced by the pCsCMV-CM and pCsCMV-NC on cassava leaves at 35 days postinoculation (dpi). **c** Detection of viral accumulation of the CsCMV-CM and CsCMV-NC in infected cassava (‘SC10’) leaves using RT-qPCR. Three independent experiments were performed and each included six plants per treatment group. Error bars indicate the SD

The full-length genomic complementary DNA (cDNA) of CsCMV-CM was obtained by PCR and then cloned between the CaMV 35S promoter (35S P) and the poly(A) signal of T-DNA binary vector pGreenII-35S [36] to generate pCsCMV-CM using Gibson assembly (Fig. 1a). To test the infectivity of pCsCMV-CM, cassava plants were infiltrated with agrobacterium-carrying pCsCMV-CM. The pCsCMV-CM was infectious and induced mild mosaic symptoms of dark and light green patches in systemically infected cassava leaves (‘SC 10’) (Fig. 1b). The CsCMV-CM genomic RNA was detected in symptomatic cassava plants agroinoculated with pCsCMV-CM but not in mock-inoculated plants by RT-PCR (Additional file 1: Fig. S1).

To create a pCsCMV-CM VIGS vector based on a strategy similar to that used to construct the PVX and FoMV VIGS vectors [32, 37], a duplicated 90-bp putative CsCMV-CM *CP* subgenomic promoter (SGP1) was added upstream of the authentic *CP* promoter (SGP2) to drive target gene expression in the context of viral RNA (Fig. 1a). The SGP1 included a 60- nt upstream and a 30-nt downstream of the *CP* start codon. Moreover, a Nimble Cloning (NC) frame sequence (adapter 1–*Sfi* I–*ccdB* gene–*Sfi* I–adapter 2) was inserted between the SGP1 and SGP2 to facilitate the rapid cloning of desired gene fragments for silencing, and the resultant vector was designated pCsCMV-NC (Fig. 1a). Agroinfiltration of cassava plants with pCsCMV-NC showed that the vector was infectious and all 18 infiltrated plants in three independent experiments developed similar mild mosaic symptoms to those of pCsCMV-CM-agroinfiltrated plants. The NC frame insertion was detected in systemic leaves by RT-PCR (Additional file 1: Fig. S1). In addition, Reverse transcription quantitative real-time PCR (RT-qPCR) revealed that there was no significant difference in the accumulation of viral RNA in cassava plants infected with CsCMV-NC compared with that in plants infected with CsCMV-CM (Fig. 1c). Thus, insertion of SGP and the NC frame into pCsCMV-CM did not affect the viral infectivity. Accordingly, each PCR-generated target fragment with NC adapters could be cloned into the pCsCMV-NC vector via Nimble Cloning.

### Silencing of cassava *PDS and ChlI* genes using the pCsCMV-NC vector

**Fig. 2:**
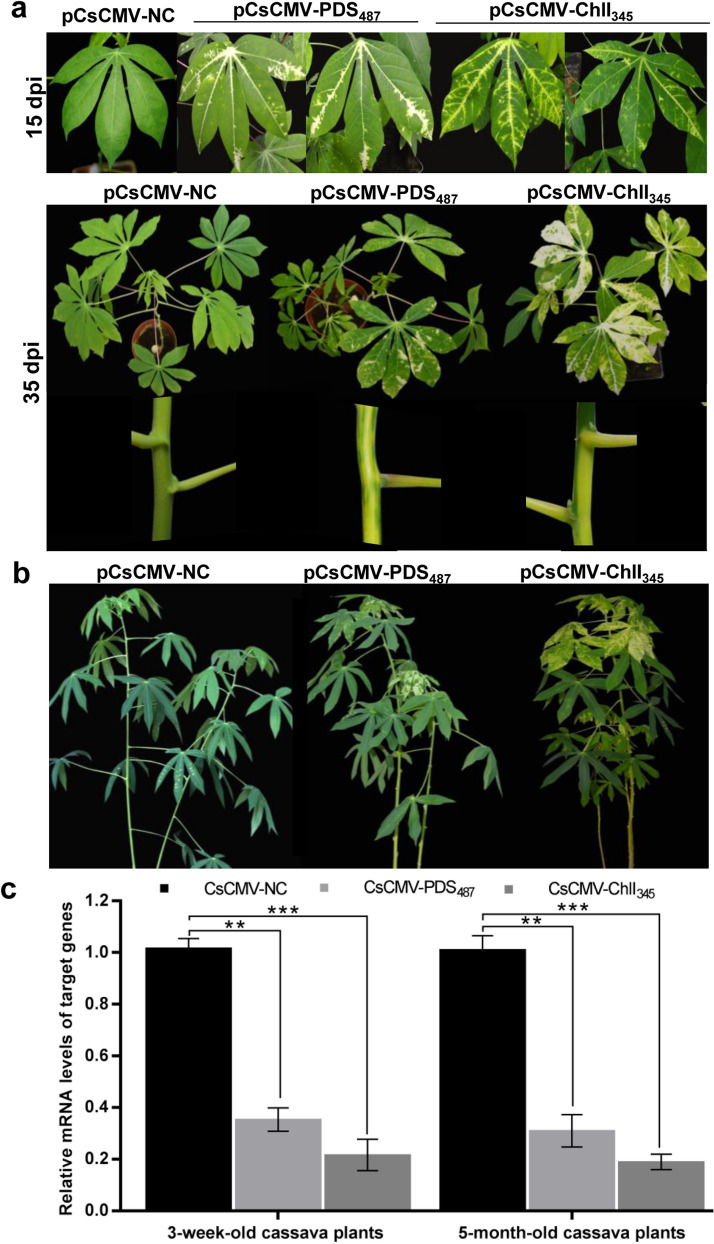
Silencing of *PDS* and *ChlI* genes in cassava using the CsCMV-based vector. **a**, Representative silencing phenotypes in 3-week-old cassava (‘SC10’) leaves and stems induced by silencing of *PDS* or *ChlI* using the CsCMV-based VIGS vector carrying a fragment of cassava *PDS* (pCsCMV-PDS_487_), *ChlI* (pCsCMV-ChlI_345_) or control vector (pCsCMV-NC) at 15 and 35 dpi. **b** Silencing phenotypes in 5-month-old cassava plants (‘SC10’) induced by infection with pCsCMV-PDS_487_ or pCsCMV-ChlI_345_ at 35 dpi. **c** RT-qPCR analyses of *PDS* and *ChlI* mRNA expression in 3-week and 5-month-old cassava plants infected with CsCMV-PDS_487_, CsCMV-ChlI_345_ and CsCMV-NC. Statistical tests were performed using Student’s *t* test, compared with plants infected with non-target control CsCMV-NC (**P < 0.01 and ***P < 0.001). Three independent experiments were performed, and each included six plants per treatment group. Error bars indicate the SD

To test whether pCsCMV-NC could be used to induce endogenous gene silencing in cassava, we first silenced two VIGS marker genes, *PDS* and *ChlI*. After genome-wide, off-target gene-silencing analysis, 487-bp *PDS* and 345-bp *ChlI* DNA fragments were cloned into pCsCMV-NC in an antisense orientation to generate the pCsCMV-PDS_487_ and pCsCMV-ChlI_345_ vectors, respectively. The 3-week-old cassava plants agroinfiltrated with pCsCMV-PDS_487_ or pCsCMV-ChlI_345_ initially exhibited mild photobleaching or a yellow-leaf phenotype in the veins of the second and third leaves above the inoculated leaves at 15 dpi, and developed severe photobleaching or a yellowing VIGS phenotype in the stems and the newly emerging leaves at 35 dpi (Fig. 2a). Similarly, the silencing phenotype occurred in the upper systemic leaves of 5-month-old cassava plants agroinoculated with pCsCMV-PDS_487_ or pCsCMV-ChlI_345_ (Fig. 2b). According to the number of leaves and areas exhibiting silencing phenotypes, silencing was more effective in the CsCMV-ChlI_345_-infected plants than in the CsCMV-PDS_487_-infected plants. The CsCMV-ChlI_345_-induced silencing phenotype could be maintained more than two months, whereas the photo-bleached leaves infected with CsCMV-PDS_487_ tended to senesce at 45 dpi (Additional file 1: Fig. S2). RT-qPCR showed that the *PDS* and *ChlI* mRNA levels were reduced by approximately 70% and 81% in the silenced leaves compared with those in the CsCMV-NC-infected leaves, respectively (Fig. 2c). These results suggest that the pCsCMV-NC vector can be used to silence endogenous genes in cassava.

### Silencing effects of different sizes of *ChlI* genes inserted into the pCsCMV-NC vector

**Fig. 3:**
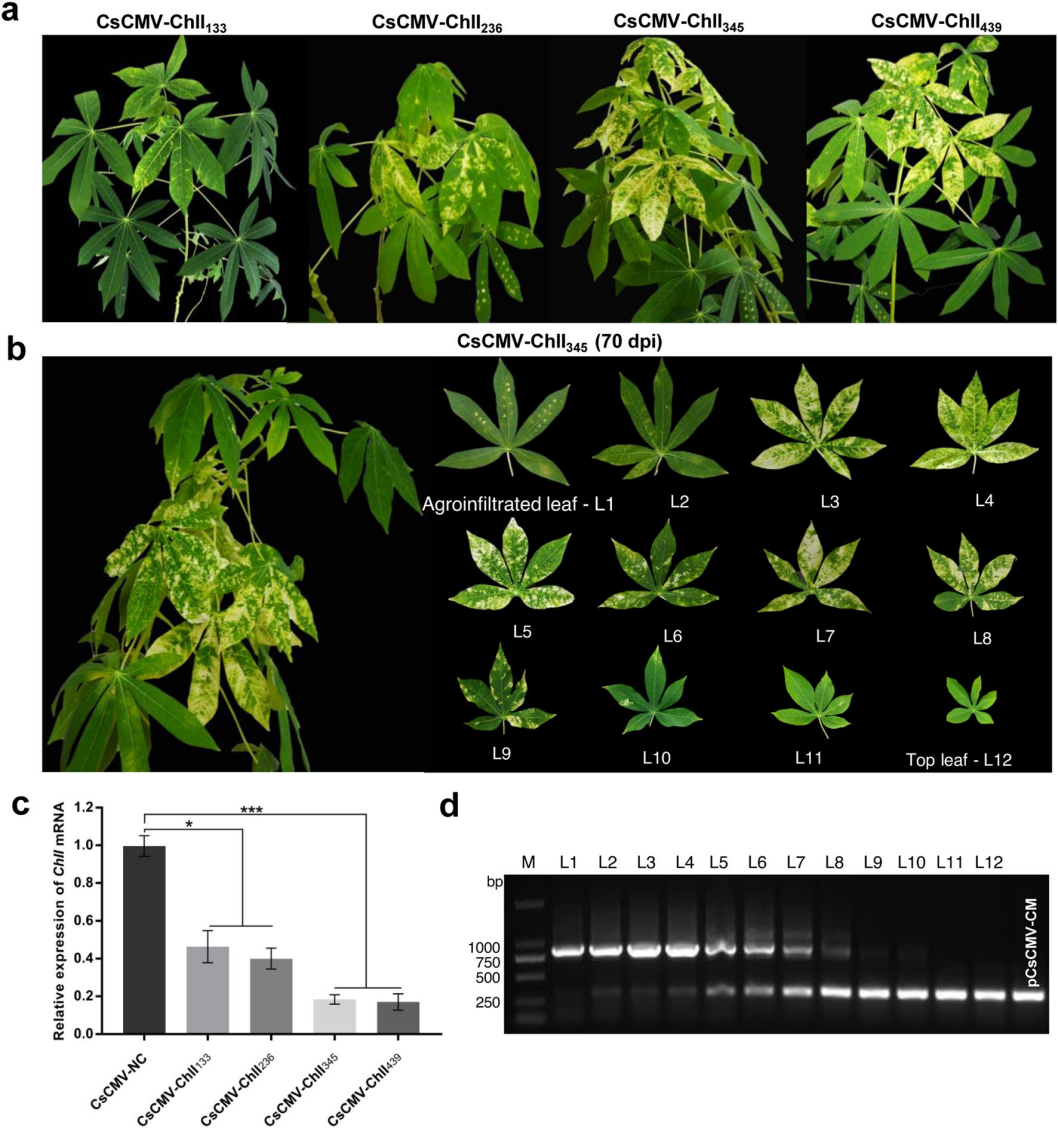
Silencing effects and stability of insert size of *ChlI* gene in the CsCMV-based VIGS vectors. **a** 5-month-old cassava plants (‘SC10’) were infected with the CsCMV -based VIGS vectors carrying different sizes (133, 236, 345 and 439 bp) of partial *ChlI* and representative silencing phenotypes by silencing of *ChlI* were photographed at 35 dpi. **b** Silencing phenotypes in the different-aged leaves induced by silencing of *ChlI* using the pCsCMV-ChlI_345_ at 70 dpi. **c** RT-qPCR analyses of *ChlI* mRNA expression in 5-month-old cassava plants infected with the CsCMV-based VIGS vectors carrying different sizes (133, 236, 345 and 439 bp) of partial *ChlI*. Satistical tests were performed using Student’s *t* test, compared with the plants infected with non-target control CsCMV-NC (*P < 0.05 and ***P < 0.001). Three independent experiments were performed and each included six plants per treatment group. Error bars indicate the SD. **d** RT-PCR analyses of the stability of the 345-bp *ChlI* fragment in pCsCMV-ChlI_345_ among the different-aged leaves. A total of 12 leaves from the agroinfiltrated leaf to the top-most leaf (L1 to L 12, numbered from the inoculated leaves) were collected for RNA extraction and RT-PCR analysis. The non-target control pCsCMV-NC was used as the control

To investigate whether the insert size affects VIGS efficiency, additional CsCMV-based VIGS constructs of different sizes carrying partial *ChlI* genes (133, 236, and 439 bp) were created. The resulting pCsCMV-ChlI_133_, -ChlI_236_, and -ChlI_439_ and CsCMV-ChlI_345_ constructs were used to separately agroinoculate 5-month-old cassava plants. During the following 30-d observation period, plants infected with CsCMV-ChlI_345_ or CsCMV-ChlI_439_ showed similar strong silencing phenotypes with larger areas of yellowing in most new leaves; a milder and unambiguous yellow-colored VIGS phenotype was observed in plants infected with CsCMV-ChlI_236_ and ChlI_133_, respectively (Fig. 3a). RT-qPCR analysis confirmed that relative expression levels of *ChlI* mRNA were reduced by 53.7%, 60%, 81.7% and 83% in the leaves exhibiting the yellow phenotype induced by CsCMV-ChlI_133_, -ChlI_236_, and -ChlI_439_ and CsCMV-ChlI_345_, respectively, compared with those in the CsCMV-NC-infected leaves (Fig. 3c). These results suggest that the insert size of target gene was related to the silencing effects.

### Stability of the 345-bp *ChlI* fragment in pCsCMV-ChlI_345_

At 70 dpi, we observed the different VIGS silencing effects in 12 leaves from the agroinfiltrated leaf to the top-most leaf (L1 to L12, numbered from the agroinfiltrated to the upper leaves) in the CsCMV-ChlI_345_-infected cassava plants. The obvious silencing phenotype was maintained in the third (L3) to eighth (L8) leaves, and the phenotype gradually became less severe in the upper leaves, almost disappearing in the top leaves (Fig. 3b). To investigate the stability of the *ChlI* fragment in pCsCMV-ChlI_345_ in the leaves of different ages, RT-PCR was carried out. It revealed that the *ChlI* insertions were relatively stable in L1 (agroinfiltrated leaf) because the expected 792-bp band was specifically amplified. However, an additional smaller band of 315 bp, whose size was similar to that of the empty pCsCMV-CM vector appeared in leaves L2 to L10 and became more obvious in the upper systemically infected leaves (Fig. 3d); this suggested that the inserted *ChlI*_345_ was partially lost to different extents in these leaves. Leaves L11 to L12 without silencing phenotypes exhibited complete deletions of the *ChlI* fragment because only the 315-bp band was detected (Fig. 3d).

### The CsCMV VIGS vector is suitable for different cassava cultivars

**Fig. 4:**
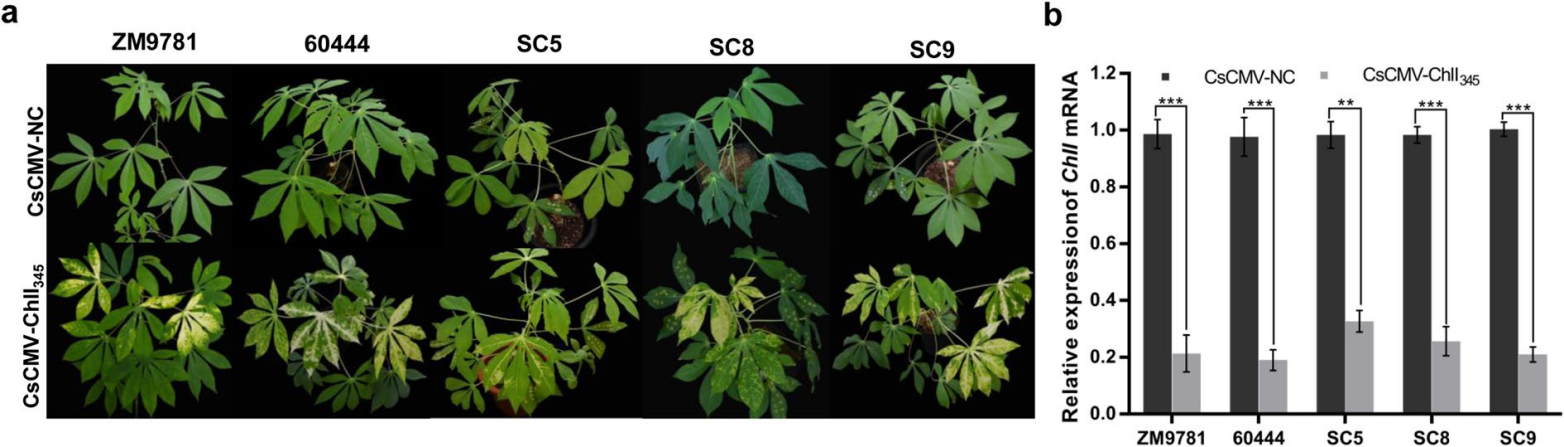
Infection and VIGS phenotypes in 5 cassava cultivars agroinfiltrated with pCsCMV-ChlI_345_ and pCsCMV-NC._._ **a** Silencing phenotypes in 5 cassava cultivars (60,444, ZM9781, SC5, SC8, and SC9) using the pCsCMV-NC and pCsCMV-ChI_345_ at 30 dpi. **b** RT-qPCR analyses of *ChlI* mRNA expression in 5 cassava cultivars infected with pCsCMV-ChlI_345_ and pCsCMV-NC. Satistical tests were performed using Student’s *t* test, compared with plants infected with non-target control pCsCMV-NC (**P < 0.01 and ***P < 0.001). Three independent experiments were performed and each included six plants per treatment group. Error bars indicate the SD

To test whether our CsCMV-NC VIGS vector could induce silencing in other cassava varieties, five additional lines popular in China (60,444, ZM9781, SC5, SC8, and SC9) were agroinfiltrated with the pCsCMV-NC and pCsCMV-ChlI_345_ vectors. All five of these lines were susceptible to pCsCMV-NC, exhibiting mild mosaic symptoms, and pCsCMV-ChlI_345_ induced the typical yellow-white phenotype to different extents in new leaves of all lines at 30 dpi (Fig. 4a). RT-qPCR showed that the mRNA levels of *ChlI* were decreased by approximately 68 to 81% in these five cultivars infected with CsCMV-ChlI_345_ compared with those in the CsCMV-NC-infected control plants (Fig. 4b).

### CsCMV-PDS_487_ and CsCMV-ChlI_345_ induced the albino and chlorotic VIGS phenotypes in *N. benthamiana*

**Fig. 5:**
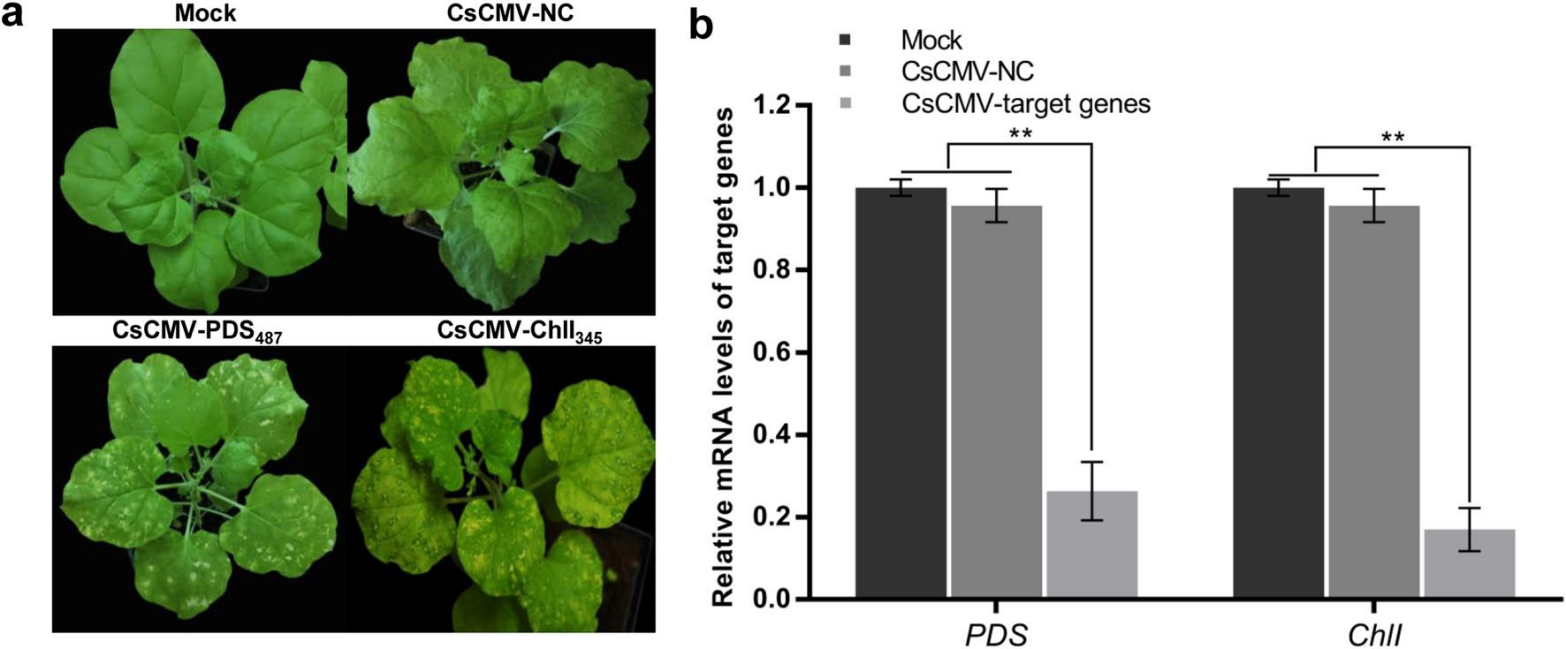
Silencing of *PDS* and *ChlI* genes in *Nicotiana benthamiana* using the pCsCMV-PDS_487_ and pCsCMV-ChlI_345._ **a** Silencing phenotypes in *N. benthamiana* using the pCsCMV-PDS_487_ and pCsCMV-ChlI_345_ at 25 dpi. **b** RT-qPCR analyses of *PDS* and *ChlI* mRNA expression in *N. benthamiana* infected with pCsCMV-PDS_487_, pCsCMV-ChlI_345_ and pCsCMV-NC. Satistical tests were performed using Student’s *t* test, compared with plants infected with non-target control pCsCMV-NC (**P < 0.01). Three independent experiments were performed and each included six plants per treatment group. Error bars indicate the SD

Like cassava, the model plant *N. benthamiana* can be systemically infected with CsCMV [25]. The 487-bp fragment of cassava *PDS* and 345-bp fragment of cassava *ChlI* in pCsCMV-PDS_487_ and pCsCMV-ChlI_345_ shared 75.7% and 82.6% similarity with *N. benthamiana PDS* and *ChlI*, respectively (Additional file 1: Fig. S3). Agroinfiltration of *N. benthamiana* with pCsCMV-PDS_487_ and pCsCMV-ChlI_345_ resulted in the appearance of photo-bleached spots and chlorosis, respectively (Fig. 5a). These silencing phenotypes could be maintained longer than two months after inoculation. RT-qPCR results showed that *PDS* and *ChlI* mRNA expression in the *N. benthamiana* plants infected with CsCMV-PDS_487_ and CsCMV-ChlI_345_ were only 26.7% and 17% of those in the CsCMV-NC-infected plants, respectively (Fig. 5b). These results suggest that the *PDS* and *ChlI* fragments from cassava could induce silencing of the corresponding orthologous genes in *N. benthamiana* via the CsCMV-based VIGS vector.

## Discussion

In this study, we developed a VIGS vector derived from CsCMV-CM for use in cassava plants. The CsCMV-based vector has several advantages over previous ACMV, EACMV, and TRV VIGS vectors in cassava [17–19, 23]. First, the vector caused milder mosaic symptoms than ACMV and EACMV in cassava leaves, making it suitable for functional genomics in cassava because severe viral symptoms may be confused with the effects of the VIGS vector and its resulting phenotypes in test plants. Second, unlike ACMV and EACMV and TRV, which consist of bipartite DNA and RNA genomes, respectively, CsCMV has a single-stranded RNA genome. A virus vector-based single viral genome is usually easier to manipulate than those of multipartite genomes [33]. For example, compared with vectors based on multipartite viruses, agroinfiltration with CsCMV vectors does not require the preparation of a mixture of agrobacterium suspensions, each one carrying discrete portions of the genome. Third, CsCMV spreads by mechanical transmission, which makes infection of plants easier through leaf agroinfiltration, resulting in effective VIGS. Conversely, agroinfiltration of cassava plants with geminivirus-based vectors requires injection of agrobacteria suspensions near the axillary buds and through superficial cuts in the stem. This inoculation method could damage the meristems and affect plant growth [19]. Biolistic delivery has also been used for geminivirus inoculation of cassava plants, but it is a high-cost method [18]. In this study, 100% infection efficiency by agroinfiltration of pCsCMV-NC vector was achieved. Fourth, ACMV and EACMV are transmitted by whitefly. Their viral vectors have a potentially higher risk of escape into the environment than the CsCMV vector by mechanical transmission. Fifth, we inserted the NC frame of Nimble Cloning into the CsCMV genome to facilitate the rapid cloning of desired target genes. Accordingly, each PCR-generated target fragment with NC adapters can be cloned into a circular pCsCMV-NC vector via a simple mixture of the rare-cutting restriction enzyme *Sfi* I and T5 exonucleases, which simultaneously accomplish linearization of the vector and the ligation reaction [35]. In previous studies, Gateway-based and ligation-independent VIGS vectors have been used for rapidly cloning a target fragment without multiple digestion and ligation steps [22, 38]. In contrast to these approaches, Nimble Cloning does not require an additional step to linearize the VIGS vector; therefore, it is simpler and more cost-effective than Gateway-based and ligation-independent cloning methods [35]. Furthermore, the NC frame includes the *ccdB* gene, which is a positive selection marker to facilitate more rapid and accurate screening of putative recombinant colonies. A similar strategy is widely applied in Gateway methods [22, 38]. In this study, greater than 95% of clones were positive in each transformation using Nimble Cloning with mixtures of pCsCMV-NC vector and PCR amplicons of individual target gene fragments. Thus, the CsCMV-based VIGS system could also be applied in the construction of a cassava VIGS library for high-throughput forward genetics screening in the future.

Application of CsCMV as a VIGS vector requires the insertion of foreign sequences into the viral genome at positions that do not affect viral infectivity. CsCMV is the type member of the genus *Potexvirus*. There are two strategies for construction of potexvirus-based vectors according to their viral genome organization. Introduction of an additional SGP upstream of the *CP* gene for expression of a gene of interest is a commonly used approach and has successfully been used in PVX, PepMV, and FoMV [31–33]. The other strategy is development of a FoMV-based VIGS vector by insertion of the cloning site after the *CP* stop codon [34]. In our study, we constructed the CsCMV vector by duplicating the 90-bp putative CsCMV *CP* SGP, including the potexvirus-specific octanucleotide motif (GUUAAGUU) [37]. Our result showed that engineering the duplicated copy of the putative CsCMV *CP* SGP and NC cloning frame into the viral genome did not affect infectivity of the CsCMV-NC vector in cassava and *N. benthamiana*. Moreover, the anti-sense fragments of *PDS* and *ChlI* cloned into the pCsCMV-NC vector caused obvious silencing phenotypes in both host plants.

In this study, the 487-bp fragment of *PDS* and the 345-bp fragment of *ChlI* with best target region score were selected by genome-wide off-target gene silencing assessment [39]. The predicted result showed that *ChlI*_*345*_-derived siRNAs can target two *ChlI* homologous genes located on the 16th and 17th chromosomes of cassava while the predicted siRNAs only can match *PDS* gene in the 5th chromosome of cassava. More target sites might result in more accumulation of the target fragment-derived siRNAs and induced more severe silencing phenotype. Indeed, we observed that the *ChlI*_345_-silenced cassava plants exhibited more severe silencing phenotype with large areas of yellow–white leaf than *PDS*_487_-silenced cassava plants. Certainly, the gene silencing efficiency is related to various factors including sequence space, target availability, the position of nucleotides, secondary structures of mRNA and intrinsic characteristics of siRNA and target mRNA [40]. In addition, we assessed whether the size of the host-derived sequence insert affects CsCMV based VIGS efficiency. Our results showed that the CsCMV-vectors carrying partial *ChlI* genes of different sizes (133, 236, 345, and 439 bp) in antisense orientation could effectively induce silencing in cassava, and the more severe silencing phenotype was observed when the insert length was more than 300 bp. Similarly, infection with the PVX VIGS vector harboring *PDS* sequences of 412-bp in antisense orientation resulted in strong photobleaching phenotypes in both diploid and cultivated tetraploid Solanum species [32]. However, FoMV vector with a duplicated FoMV CP SGP was used to induce effective silencing of endogenous genes in barley when target sequence insert was a short inverted-repeat fragment but not an antisense one [33]. Therefore, the effect of length of target genes on silencing depends on the different potexviruses-derived vectors and hosts.

As VIGS approaches induce transient gene knockdowns, increasing the duration of endogenous gene silencing will widen the application of VIGS in functional genomics. In this work, strong yellow-white silencing phenotype in systemic leaves infected with CsCMV-ChlI_345_ can persist for more than two months. The longer silencing period will facilitate characterization of the gene functions involved in developmental and biosynthetic pathways and stress tolerance in cassava. However, the phenotype gradually became less severe in the upper leaves and almost disappeared in the top leaves, which was related to partial or complete loss of inserted *ChlI*_*345*_ fragment because of the sequence redundancy of the duplicated SGP in potexvirus-based vectors [34, 37]. To address the problem, an PVX-based expression vector was improved to stabilize the foreign inserts by replacing the duplicated SGP with a heterologous SGP combined with an N-terminal CP deletion [37]. In addition, change of the position of insertion was used to increase stability of the insert. The cloning site in FoMV was placed after the stop codon following the CP coding sequence instead a duplicated subgenomic promote [34]. However, the loss of *PDS* inserts still occurred when this FoMV vector was used to silence *PDS* in maize [34]. Therefore, the stability of sequences inserted into viral genomes is regard as a surprisingly complex problem involved in the genome characteristics, the host environment and the demography of a virus population [41].

Over the years, numerous cassava varieties with different traits have been released in the world [2]. Theoretically, CsCMV-based VIGS system are applicable to cassava lines susceptible to CsCMV. Here, we induced *ChlI* gene silencing in 6 popular lines (60,444, ZM9781, SC5, SC8, SC9 and SC10) in China using pCsCMV-ChlI_345_, which will contribute to use this vector to analysis some functional genes involved in important biological and agronomical traits among these cultivars. In addition, CsCMV were detectable in fibrous and storage roots of CsCMV-NC-infected cassava plants (Additional file 1: Fig. S4), thereby we will further broaden the use of CsCMV vector in gene silencing from leaves to root tissues like ACMV-based vector.

## Conclusions

We developed an effective CsCMV-based VIGS vector that induced endogenous gene silencing in different cassava cultivars. Target fragments for gene silencing can easily be cloned into the CsCMV vector using one-step Nimble cloning. The new VIGS system will facilitate rapid and high-throughput loss-of-function studies in cassava.

## Methods

### Generation of a CsCMV agroinfectious clone

Total RNA was extracted from the CsCMV-infected cassava leaves displaying mild mosaic symptoms in a germplasm garden in Chengmai (CM) of Hainan Province, China. The first-strand cDNA was synthesized from 1.0 µg of total RNA with the Takara RNA PCR Kit (AMV) Ver. 3.0 (TaKaRa, Japan) using random 9 mers and oligo dT-Adaptor primers. The complete genome sequence of the CsCMV isolate, designated CsCMV-CM, were determined by RT-PCR and SMARTer 5′/3′ RACE kits (TaKaRa) based on our recent study [25], respectively. All primers used for PCR amplification of viral genome are listed in Additional file 2: Table S1. This whole genome sequence of CsCMV-CM has been deposited in GenBank under the accession number MW175326.

The full-length viral sequence and the backbone fragment of pGreenII-35S vector were individually PCR-amplified using CsCMV-CM cDNAs and pGreenII-35S plasmid [36] as templates with two primer pairs CsCMV-5Fov/CsCMV30T-R and CsCMVpGr-F/pGr35S-R which shared 25–36 homologous bases at each end (Additional file 2: Table S2). Then, both overlapping PCR products were mixed and assembled to generate pCsCMV-CM according to the instructions of Gibson Assembly Cloning Kit (NEB, USA). Briefly, 100 ng each purified PCR fragment and 5 µL 2 × Gibson mix (NEB) was incubated at 50 °C for 1 h, and then placed on ice for *Escherichia coli* strain DH5α transformation. The resultant clones were confirmed by PCR with primer pair CsCMV5259F/CsCMV3R and DNA sequencing. Similarly, three overlapping DNA fragments (I, II, and NC) were amplified in separate PCRs to construct the pCsCMV-NC. The DNA fragment I containing the *replicase,* the *TGB* genes and the duplicated 90-bp putative CsCMV-CM *CP* SGP1 was amplified from pCsCMV-CM using the primers CsCMV-5Fov/NC-CsCMV5623-R. The SGP1 contained the 60-nt upstream and 30-nt downstream of the CP start codon. The pCsCMV-CM was used as the template with the primers NC-CsCMV5534-F/pGr35S-R to amply the DNA fragment II covering the authentic CP promoter SGP2 and the backbone fragment of pGreenII-35S. The NC frame from pNC-UC vectors [35] were amplified using primer pairs NCF/NCR. All primer pairs used Gibson Assembly included sequences overlapping adjacent fragments by 21 to 25 nt. The Gibson Assembly reaction of pCsCMV-NC is same as described for the construction of pCsCMV-CM. The transformation was performed using *Escherichia coli* strain DB3.1.

### Generation of CsCMV VIGS constructs

The regions of target genes for genome-wide off-target gene silencing were selected using SGN VIGS Tool [39]. A 487 bp cassava *PDS* fragment of (GenBank accession: XM_021757403) and four partial cassava *ChlI* fragments (GenBank accession: XM_021743433) of different sizes (133, 236, 345 and 439 bp) was amplified using cassava cDNAs as a template and the corresponding primer pairs (Additional file 2: Table S3). Then the amplified fragments were cloned into pCsCMV-NC to generate pCsCMV-PDS_487_, pCsCMV-ChlI_133_, -ChlI_236_, -ChlI_345_ and -ChlI_439_ using Nimble Cloning [35]. In brief, 20–100 ng circular destination vector (1–2 µL) and 10–50 ng PCR insert were added a PCR microtube containing 5 µL 2 × Nimble Mix for a final volume of 10 µL. The reaction mixture was incubated in a water bath for 1 h at 50 °C and then performed transformation in *Escherichia coli* strain DH5α. The accuracy of all resulting constructs was identified by sequencing.

### Plant growth and agroinfiltration

Cassava plants were propagated vegetatively by planting properly lignified stem cuttings in soil. Cassava and *Nicotiana benthamiana* plants were grown in a greenhouse at 25 °C under a 16/8-h photoperiod. 3-week and 5-month-old cassava plants, and 2-week-old *N.benthamiana* seeding were used for agroinoculation. For agroinfiltration of recombinant CsCMV clones, the CsCMV-NC-based constructs were transformed into *Agrobacterium tumefaciens* GV3101 with pSoup helper plasmid, respectively. A single colony of *A. tumefaciens* strain GV3101 for each viral construct were gown overnight in Luria–Bertani medium containing rifampicin (25 mg/L) and kanamycin (50 mg/L) at 28 °C. Subsequently, overnight bacterial cultures were centrifuged at 2,500*g* for 10 min and were resuspended in agroinfiltration buffer (10 mM MgCl_2_, 10 mM 2-(N-Morpholino) ethanesulfonic acid [pH 5.5], and 100 µM acetosyringone) for reaching an optical density of 0.8 at 600 nm (OD_600_) [42]. The agrobacterium suspension was kept at room temperature for 3 h in the dark before agroinfiltration. For cassava, the back sides of four healthy and fully developed leave in the middle of each plant were selected for agroinfiltration using a 1-mL needleless syringe. Injections were performed at 8–10 spots on both sides of the main vein per leaf to enlarge the infiltrated leaf area. About 10 µL of agrobacterium suspension was used for each spot. *N. benthamiana* leaves were agroinfiltrated as described previously [43].

### RT-PCR and RT-qPCR analysis

Total RNA of cassava was extracted using the RNAprep Pure Plant Kit (Tiangen Biotech, China). For RT-PCR, the first-strand of cDNAs from 1.0 µg of total RNA were synthesized with the Takara RNA PCR Kit (AMV) Ver. 3.0 (TaKaRa) using random 9 mers and oligo dT-Adaptor primers. To test the stability of the inserted target fragments in CsCMV-based vectors during viral infection, RT-PCR was performed using the primer pair CsCMV5416F(5′-TTGTAGCTGCCGTCCTAACTTGG-3′) /5730R (5′-ACCAAATTGGAGGCTGGCTTCA-3′) flanking the NC frame. For RT-qPCR, three independent experiments were performed and each included six plants per treatment group. Two to three systemic leaves with symptomatic or silencing phenotypes in cassava plant agroinoculated with each CsCMV construct were pooled for total RNA extraction. The cDNAs from 1 μg of DNA-free RNA and oligo (dT) using PrimeScript RT Reagent Kit (TaKaRa) were synthesized following the manufacturer’s instructions. All RT-qPCR reactions were carried out using SYBR Premix EX Taq II Kit (TaKaRa). The cassava *Mepp2A* gene and *N. benthamiana actin* gene were used as an internal control for normalizing the expression of target genes. The species-specific primer pairs for *PDS* and *ChlI* (Additional file 2: Table S4) were used to test the silencing effect in each of these genes, and the expression level of each target gene was calculated using the delta-delta Ct method compared with the expression levels of the corresponding gene in the CsCMV-NC-infected samples [25]. The accumulation levels of CsCMV-CM and CsCMV-NC in systemically infected plants were quantified using the specific primers of CsCMV coat (*CP*) gene (Additional file 2: Table S4).

### Statistical analyses

Statistical analyses were performed using SPSS 23.0 (SPSS Inc., Chicago, IL, USA) software and all graphs were generated by Origin 2019 (OriginLab Corporation, Northampton, MA, USA). Statistical significance was assessed using Student’s *t* test, values of *P* < 0.05 were considered statistically significant. Data were presented as the means ± standard deviation (S.D.) of three biological replicates.

## Supplementary Information

**Additional file 1: Figure S1.** Detection of viral RNA of the CsCMV-CM and CsCMV-NC in infected cassava leaves using RT-PCR. **Figure S2.** Silencing phenotypes in 5-month-old cassava plants induced by infection with pCsCMV-PDS_487_ or pCsCMV-ChlI_345_ at 45 dpi. **Figure S3.** Alignment of the cassava 487-bp *PDS* (GenBankaccession:XM_021757403) and 345-bp *ChlI* gene fragments(GenBankaccession:XM_021743433) in pCsCMV-PDS_487_ and pCsCMV-ChlI_345_ with the *N.benthamiana* *PDS* (GenBankaccession:DQ469932) and *ChlI* (SequenceID:Niben101Scf16898g00001.1) homologues by the Sol Genomics Network (SGN). (https://solgenomics.net/organism/Nicotiana_benthamiana/genome). **Figure S4.** Detection of viral RNA of the CsCMV-CM and CsCMV-NC in cassava storage roots using RT-PCR.

**Additional file 2: Table S1.** Primers used in identification of the complete genome sequence of the CsCMV-CM.** Table S2.** Primers used in construction of agroinfectious clone pCsCMV-CM and pCsCMV-NC vector.** Table S3.** Primers used for Nimble Cloning of gene fragments into the pCsCMV-NC vector.** Table S4. **Primers used for quantitative RT-PCR analyses.
