# Supplementary material for: A cassava common mosaic virus vector for virus-induced gene silencing in cassava
Source: Plant Methods. 2021 Jul 12;17:74. doi: 10.1186/s13007-021-00775-w (PMC8273954; doi:10.1186/s13007-021-00775-w)
Supplement: Supplementary file 1 — Additional file 1: Figure S1. Detection of viral RNA of the CsCMV-CM and CsCMV-NC in infected cassava leaves using RT-PCR. Figure S2. Silencing phenotypes in 5-month-old cassava plants induced by infection with pCsCMV-PDS487 or pCsCMV-ChlI345 at 45 dpi. Figure S3. Alignment of the cassava 487-bp PDS (GenBankaccession:XM_021757403) and 345-bp ChlI gene fragments(GenBankaccession:XM_021743433) in pCsCMV-PDS487 and pCsCMV-ChlI345 with the N.benthamiana PDS (GenBankaccession:DQ469932) and ChlI (SequenceID:Niben101Scf16898g00001.1) homologues by the Sol Genomics Network (SGN). (https://solgenomics.net/organism/Nicotiana_benthamiana/genome). Figure S4. Detection of viral RNA of the CsCMV-CM and CsCMV-NC in cassava storage roots using RT-PCR. [file 13007_2021_775_MOESM1_ESM.pdf]

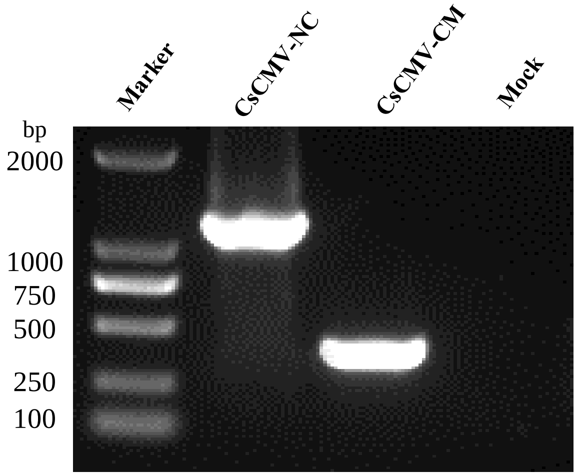

Figure S1: Detection of viral RNA of the CsCMV-CM and CsCMV-NC in infected cassava leaves using RT-PCR

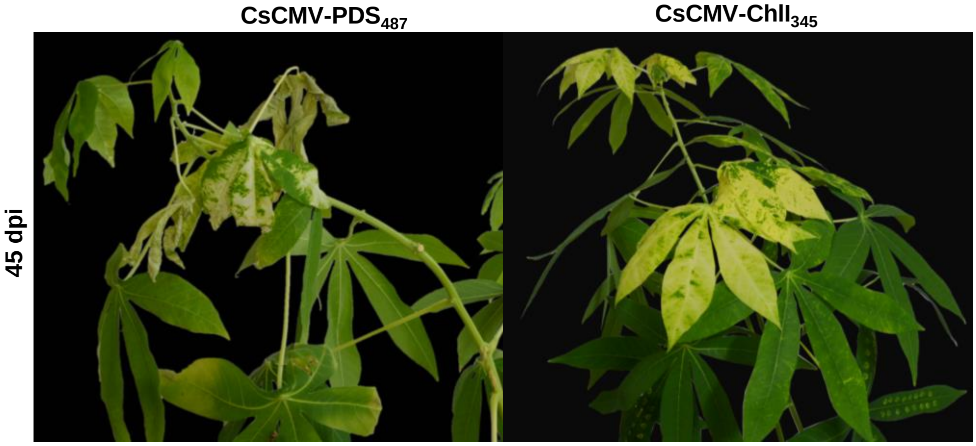

Figure S2:Silencing phenotypes in 5-month-old cassava plants induced by infection with pCsCMV-PDS$_{487}$ or pCsCMV-ChlI$_{345}$ at 45 dpi.

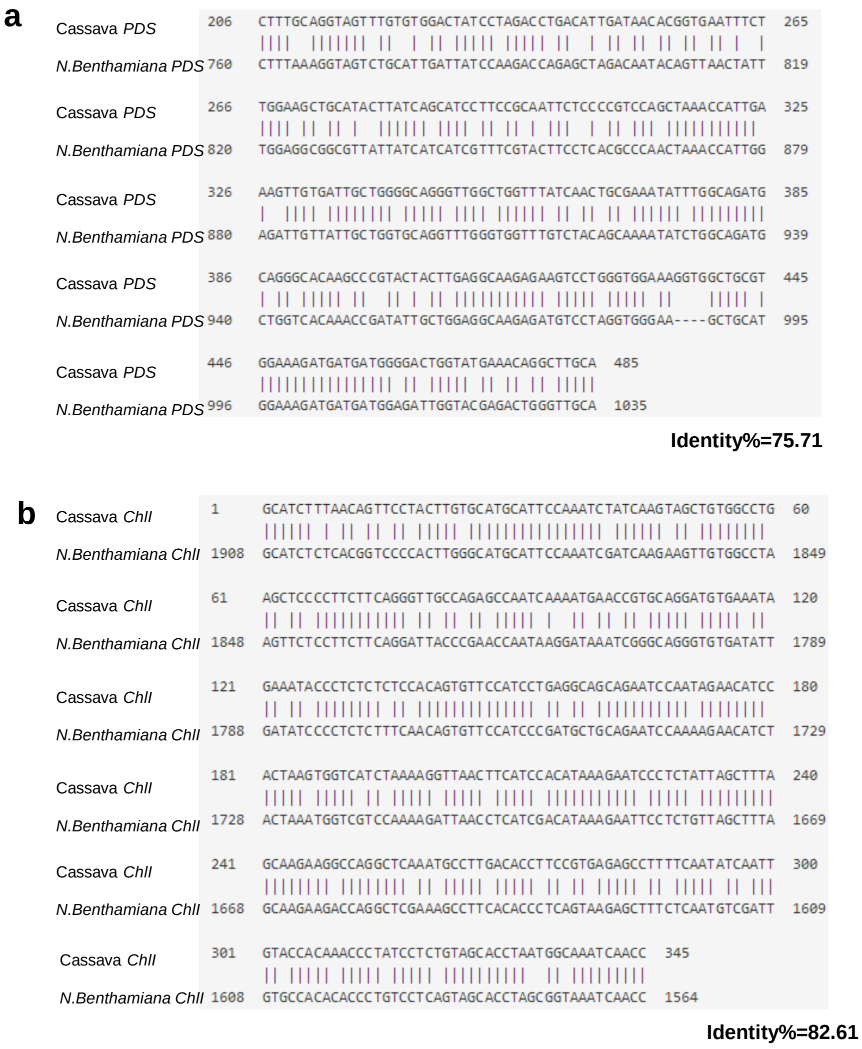

Figure S3: Alignment of the cassava 487-bp *PDS* (GenBank accession: XM_021757403) and 345-bp *ChlI* gene fragments (GenBank accession: XM_021743433) in pCsCMV-PDS$_{487}$ and pCsCMV-ChlI$_{345}$ with the *N. benthamiana PDS* (GenBank accession: DQ469932) and *ChlI* (Sequence ID:Niben101Scf16898g00001.1) homologues by the Sol Genomics Network (SGN). (https://solgenomics.net/organism/Nicotiana_benthamiana/genome).

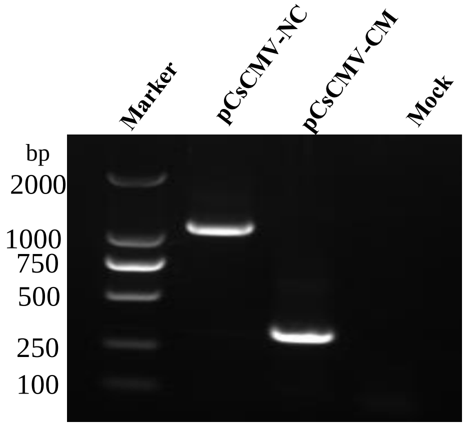

Figure S4 :Detection of viral RNA of the CsCMV-CM and CsCMV-NC in cassava storage roots using RT-PCR.
