# Supplementary material for: A cassava common mosaic virus vector for virus-induced gene silencing in cassava
Source: Plant Methods. 2021 Jul 12;17:74. doi: 10.1186/s13007-021-00775-w (PMC8273954; doi:10.1186/s13007-021-00775-w)
Supplement: Supplementary file 2 — Additional file 2: Table S1. Primers used in identification of the complete genome sequence of the CsCMV-CM. Table S2. Primers used in construction of agroinfectious clone pCsCMV-CM and pCsCMV-NC vector. Table S3. Primers used for Nimble Cloning of gene fragments into the pCsCMV-NC vector. Table S4. Primers used for quantitative RT-PCR analyses. [file 13007_2021_775_MOESM2_ESM.pdf]

Table S1. Primers used in identification of the complete genome sequence of the CsCMV-CM

Primer	Sequence (5′-3′)	Application
CsCMV5F	GGAAAACCTCACATTCCAAACCAAAAC	Full-length cDNAs of CsCMV
CsCMV3R	ATACCGCTAAACTGTCCGCGGG	
CsCMV448R	GTTGATGAAGTGGTCACGGTGTTG	5´RACE
CsCMV5259F	GGACCAAGACTGTTTACTATG	3´RACE

Table S2. Primers used in construction of agroinfectious clone pCsCMV-CM and pCsCMV-NC vector

Primer	Sequence (5′-3′)	Application
CsCMV-5Fov	AGGAAGTTCATTTCATTTGGAGAGGGGAAAACCTCACATTCCAAACCAAAAC	pCsCMV-CM
CsCMV30T-R	CGATTTTTTTTTTTTTTTTTTTTTTTTTTTTTTTATACCGCTAAACTGTCCGCGGG	
CsCMVpGr-F	TATAAAAAAAAAAAAAAAAAAAAAAAAAAAAAAATCGGTACGCTGAAATCACCAGTCT	pCsCMV-CM
pGr35S-R	CCTCTCCAAATGAAATGAACTTCCT	
CsCMV-5Fov	AGGAAGTTCATTTCATTTGGAGAGGGGAAAACCTCACATTCCAAACCAAAAC	pCsCMV-NC
NC-CsCMV5623-R	AGGACTGGACAGAGACCACTGTGTAGGAGTGGTTGAGGTGGGGGTGGCCCTAGCTAATT	(fragment I)
NC-CsCMV5534-F	ACTTGTGGTCTGCTGAGACCAAGAGATTTTGGCAAACCTCCATCC	pCsCMV-NC
pGr35S-R	CCTCTCCAAATGAAATGAACTTCCT	(fragment II)
NC-F	CAGTGGTCTCTGTCCAGTCCT	pCsCMV-NC
NC-R	TGGTCTCAGCAGACCACAAGT	(fragment NC)

Underlined sequences corresponding to the overlapping region used Gibson Assembly.

Table S3. Primers used for Nimble Cloning of gene fragments into the pCsCMV-NC vector.

Gene	GenBank Accession number	Size (bp)	Position	Primer sequence (5′-3′)
PDS (Cassava)	XM_021757403	487	293-779	AGTGGTCTCTGTCCAGTCCTTATGCAAGCCTGTTTCATACCAGTC<br>GGTCTCAGCAGACCACAAGTACTTTGTACGGGAGTGTTTCTGCAT
ChlI (Cassava)	XM_021743433	439	545-983	AGTGGTCTCTGTCCAGTCCTGCATCTTTAACAGTTCCTACTTGTG<br>GGTCTCAGCAGACCACAAGTCAGATCCAGAAGATCCAGAATCCA
		345	639-983	AGTGGTCTCTGTCCAGTCCTGCATCTTTAACAGTTCCTACTTGTG<br>GGTCTCAGCAGACCACAAGTGGTTGATTTGCCATTAGGTGCTAC
		236	748-983	AGTGGTCTCTGTCCAGTCCTGCATCTTTAACAGTTCCTACTTGTG<br>GGTCTCAGCAGACCACAAGTGCTAATAGAGGGATTCTTTATGTGGATG
		133	851-983	AGTGGTCTCTGTCCAGTCCTGCATCTTTAACAGTTCCTACTTGTG<br>GGTCTCAGCAGACCACAAGTGAGAGGGTATTTCTATTTCACATCCTGC

Underlined sequences corresponding to the adapter 1 and 2 of Nimble Cloning

Table S4. Primers used for quantitative RT-PCR analyses

Gene	GenBank Accession number/ Sequence ID	Primer sequence (5′-3′)
PDS (Cassava)	XM_021757403	GTTTGCTACTCCAGTTGACATC<br>AGTTTCCTGTCGAACCATATGT
ChlI (*Cassava)*	XM_021743433	GCTGAGCTGAATGTGGATGG<br>GGGATAACAGTAGCAACGTCCT
Mepp2A (Cassava)	Manes.09G039900	TGCAAGGCTCACACTTTCATC<br>CTGAGCGTAAAGCAGGGAAG
PDS (*Nicotiana benthamiana*)	DQ469932	CTTATGTTGAAGCTCAAGACGG<br>AATATGTTCCACAATCGGCATG
ChlI (Nicotiana benthamiana)	Niben101Scf16898g00001.1	GCAGAGTCAAGGGAGGAAGT<br>TGGCTCTCCTCAGCAAGTTT
Actin (*Nicotiana benthamiana*)	AY179605	GGCATTCATGAAACCACATACA<br>AGGACAATGTTTCCGTACAGAT
CP (CsCMV)	MW175326	GCCCTAGACCTGGTCAACTT<br>AGCAGGAGGTATGTTGGCTT
